# 

**Published:** 1890-11-01

**Authors:** 


### NOTICE TO CORRESPONDENTS.

All MS., Letters, Books for Review, and other matters intended for the Editor should be addressed The Editor, The Lodge, Porchester Square, London, W.

The Editor cannot undertake to return rejected M.S., even when accompanied by stamped directed envelope.

**Notice to Advertisers and Subscribers.**

All Advertisements, Orders for Copies of The Hospital, and Business Communications should be addressed to The Publisher, *not to the Editor*, The Hospital, 140, Strand, W.C.

**Bound Volumes of "The Hospital."**

Vol. VI., containing full page portraits of T.R.H. the Prince and Princess of Wales, and Miss Florence Nightingale, is still obtainable.

Vol. VII. and all the earlier volumes are also for sale.

Orders will be received for Vol. VIII., 4s. post free. Cases for binding may be had of the Publisher, at 1s. 6d. each.

**To Residents, Secretaries, and Matrons.**

The Editor will be glad to have the Regulations and each Report as issued by Asylums, Hospitals, Convalescent and Nursing Institutions, as well as those of other Charities, and an early copy in duplicate of all Appeals and Festival Notices, etc., addressed to *The Editor, The Lodge, Porchester Square, London, W.* Any superintendent, secretary, matron, or other chief official who regularly sends such information may be registered, on application to the Publisher, to receive free of cost a cover for binding The Hospital in half-yearly volumes.

### BURDETT'S HOSPITAL ANNUAL FOR 1890

May be obtained of the Publisher, The Hospital Offices, 140, Strand, W.C., price 2s. 6d. limp boards, or 3s. 6d. cloth. All orders must be accompanied by a remittance.

### GENERAL CHARITIES.

[This Column is devoted to an account of work of charitable institutions other than Medical Charities. The Editor will be glad to receive reports or news of work at 140, Strand. *Philanthropy* should be plainly written on the envelope.]

MISS ASHTON, of Fallowfield, at the request of Mrs. Moorhouse, wife of the Bishop of Manchester, has consented to act as diocesan associate in the diocese of Manchester for domestic economy of **THE GIRLS' FRIENDLY SOCIETY.**

**THE CONVALESCENT DINNERS SOCIETY** resumed work on the first Thursday in October, having been closed, as usual, from the first Thursday in July. Hon. secretary, Miss Carbonell, 114, Queen's Gate, S.W.

A branch of **THE YOUNG WOMEN'S CHRISTIAN ASSOCIATION** has been formed at Carrbridge, in Scotland, and during the past week a conference of Somersetshire, Gloucestershire, Clifton, and Bristol secretaries, referees, and workers has been held at Weston-super-Mare.

Among the numerous institutions which have to make periodical appeals for support, and which unfortunately have to call attention to a falling off of donations is **THE SOLDIERS' DAUGHTERS' HOME** at Hampstead. Not only have the donations and subscriptions fallen off, but the amount paid for boarders has during the last financial year been less than usual. The Committee of Management account for the decrease in annual subscriptions by some of the regiments having ceased to contribute their annual subscription. The reason which has prompted certain regiments to take this step is not announced, but considering the benefit conferred by the institution on the army at large it is certainly a regrettable fact. During the year twenty-nine girls were admitted to the home, and the average number of girls in the home was 168, the average cost per head amounting to £21 5s. 8½d. This cost includes all expenses, with the exception of the cost of outfits for girls leaving the home, on which a sum of one hundred and forty pounds was expended. We are glad to find that the diet of the girls, on the sufficiency of which some doubt had been expressed, has been improved. The addition of half-an-ounce of dripping for each girl with her bread at tea-time cannot be a great strain on the resources of the institution, but it appears to have had the desired effect of adding to the happiness and health of the children. Over 1,000 girls have been admitted to the home since it was first opened in 1855. Of these 530 have been placed in domestic service on leaving the home, and 24 have been appointed schoolmistresses and teachers. Before many weeks are past the Christmas school treat will take place, and any contributions which will enable the committee to add to the happiness of the young lives under their charge will be gratefully acknowledged by the secretary, Mr. C. R. Low, 5, Robert Street, Adelphi, W.C.

**THE HOME TEACHING SOCIETY FOR THE BLIND** has secured the services of Mr. Frank Bevan as treasurer and trustee in the room of his father, the late Mr. R. C. L. Bevan.

**THE NATIONAL INDUSTRIAL HOME FOR CRIPPLED BOYS** at Wright's Lane, Kensington, takes in lads between the ages of thirteen and eighteen for three years on a payment of £10 a-year, and teaches them during that time to become either carpenters, saddlers, tailors, or relief stampers. The boys must be capable of using their hands, they must not be at all mentally afflicted, and must not bear a bad character or have been convicted of crime. We were very glad to see for ourselves that the training the boys get in their various trades is thoroughly workmanlike and practical. Those least crippled were making harness all by hand, or were carpentering and turning out really well-fitting bookshelves, cupboards, tables, &c. Carpentering is very favourite work, and many choose it as the trade they prefer, and then, poor fellows, find themselves physically incapable for it. A bright set were doing copper-plate printing, book-binding, and die sinking. Those most crippled were sitting on a large table, stitching away at all sorts of clothes, some of these tailors were quite crippled in their legs and back, only able just to move a little to one side or the other, but looking for the most part happy; as though life was not altogether a bad thing when hands and brains remained to give useful occupation. There is a great big playground and a reading-room, where the boys may go after they leave off work at five. There is no voting for election at this home, priority of application by the patron or person who guarantees the three years' payment alone decides the admission, subject, of course, to the necessary regulations. There are very few of these cripples' industrial schools, and their work deserves to be recognised as an immense good; they turn out the inmates as useful members of society, able to rely on their own handiwork for a livelihood, whereas the uneducated and neglected cripple of poverty is a misery to himself, and probably his only chance of gaining existence is the public display of his physical infirmity. Orders for visiting cards, or bookbinding, or any of the industries we have mentioned are gratefully received by the Resident Secretary, Mr. Bovis.

### SCRAPS AND GLEANINGS.

A MATTHEWS DUNCAN MEMORIAL is to be raised in Aberdeen.

THE students and nurses of the London Hospital sent a wreath to the funeral of Madame Anna Williams.

MR. COMPTON'S dramatic company give a matinée at Portsmouth on November 8th in aid of the hospital.

A BAZAAR has been held this week at Crouch Hill in aid of the Homes for Aged Christian Blind Men and Women.

A BRILLIANT concert has been held at Belfast in aid of the Ulster Hospital for Consumption and Diseases of the Chest.

THE Duke of Clarence and Avondale opened the Liverpool Royal Infirmary on Wednesday. Full particulars next week.

NEWCASTLE held its twenty-first Hospital Sunday this week; and there are still towns which have never yet held a Hospital Sunday!

SIR EDWIN SAUNDERS has accepted the treasurership of the Dental Hospital of London, in the place of the late R. C. L. Bevan, Esq.

A MAN advertises for a competent person to undertake the sale of a new medicine, and adds that it will prove highly lucrative to the undertaker.

THE ladies of Worcester have had a very successful Hospital Saturday collection. Lady Mary Douglas and the Mayoress (Mrs. Claughton) were amongst the collectors.

LAST Sunday the annual collection in aid of the hospitals was held at Birmingham. It is hoped that the result will be £5,000 for the Queen's Hospital, which is in special need of help.

LORD DERBY has intimated to the Town Clerk of Bury that he will give £1,000 towards providing gymnastic appliances in the two recreation grounds in the Walmesley Road and Manchester Road in that town.

THE Mayor of Liverpool presided at the annual meeting of the St. Paul's Eye and Ear Hospital, and appealed to the ladies and gentlemen of Liverpool for increased support. Dr. Walker remarked that they had often to refuse patients because there was no room for them.

THE medical officer of Swindon sends a concise and sensible report to the Local Board, praising the Wroughton Isolation Hospital, and pointing out the evils of floods such as that which happened in July, and which could be averted by further provision for carrying off storm water.

THE recent increase in the number of cases requiring admission to asylums in Dorset has necessitated the enlargement of the existing system, and the County Council have agreed upon a scheme that has been laid before them, which will provide 200 additional beds, and involve an outlay of from £60,000 to £70,000.

THE Lord Chancellor of Ireland attended a meeting of the County Galway Infirmary on October 22nd, which was called for the purpose of electing a surgeon in place of Dr. Brown, deceased. There was not a quorum of life governors present, and the meeting was dissolved. Evidently they don't "enthuse" on the hospital question in Co. Galway!

PETER WILLIAMS, described as a native of Holywell, North Wales, and who states that he is 100 years of age, was on Saturday admitted into Swansea Workhouse in a destitute and starving condition.

FOUR serious accidents in the football field occurred in Sheffield on Saturday. Arthur Bentley had his right leg broken by being knocked down. Arthur Shaw, in another match, met with a similar accident; Frank Spooney, in a match against Rotherham Club, had his collar bone broken; and John Push had his arm broken. All the men were taken to Sheffield Infirmary.

AT a special convocation of Dublin University on Tuesday, the honorary degree of D.C.L. was conferred on Mr. H. M. Stanley and Surgeon Thomas Parke, of the "Emin Relief" Expedition. Mr. Stanley was not present. Dr. Parke attended, and was introduced by Dr. Phillipson. The services of the two explorers were highly eulogised by members of the senate.

THE West of England Eye Infirmary treated over 2,000 patients last year. The committee, in their report, regretted the loss which the institution had sustained through the death of Mrs. Saturley, the late matron, and reported that Miss Kinninmont, who had been trained at the Leeds General Hospital and the London University College Hospital, had been appointed to the post.

DR. GEORGE W. LLOYD, assistant superintendent at the Flatbush Insane Asylum, Brooklyn, was shot dead on the 9th inst. by James D. Doherty, lately an inmate of the asylum, but who had escaped, and had returned there on the day mentioned armed with two revolvers. Doherty is a half-witted man, and was supposed to be harmless. He was locked up in the asylum for persistently annoying Miss Mary Anderson.

THE Duke of Portland, on the 27th inst., opened at Mansfield a new hospital which has been erected in commemoration of her Majesty's Jubilee at a cost of £3,000. His grace gave a cheque for £300 to the endowment fund, and asked to be allowed to contribute the cost of one bed in the hospital on behalf of his wife. The duchess, he stated, was much better than before she went to Scotland, and she hoped in a short time to take part in public duties.

BRIGHTON Hospital Saturday Fund has been distributed as follows:—Sussex County Hospital, £500; Brighton and Hove Dispensary, £160; Alexandra Hospital, £150; the Women's Hospital, £75; the Provident Dispensary, £35; Throat and Ear Dispensary, £30; the Eye Hospital, £30; the Dental Hospital, £30; Homœopathic Dispensary, Middle Street, £20; the Medical Mission, £10; the Nursing Association, £10; St. Mary's Home Dispensary, £10.

### The Student of Medicine.

[All communications for this Supplement should be addressed to The Editor of The Hospital, at 140, Strand, with the words, "Students' Column," written in the left hand top corner of the envelope.]

### EDITORIAL.

"Work while you are young, the world is ruled more by young men now than it used to be." These words of sound advice, and full of meaning, were uttered by one of the lecturers in the opening address to the students at one of the leading London hospitals a few days ago. In presenting to the students of medicine (we prefer this term to that more commonly in vogue, namely, medical students), the first number of our fresh, and we may say novel, attempt to provide them with a weekly journal, in which to give to the world at large their views on many subjects of divers interest, we think that these words, as a motto, come in most appropriately, both as a ground to work upon, and as a way of presenting to the rising generation of students of the science and practice of medicine, that they together made up a very important body. A student to-day, and member of the leading profession to-morrow, each one has an enormous power to help forward the rapid progress of the race—a civilisation such as ours at the present day, one which has made enormous strides within a very few years, is mainly, if not entirely, due to the unselfish earnestness of workers in the various fields or branches of the natural sciences. For countless generations we were hampered with the most grotesque superstitions, and haunted with the most ridiculous sentiments; now that the voice of science is being heard in our midst, old things have passed away, and all things have become new. On purely scientific lines we intend to publish matters of general and of special interest to the students, to give them an opportunity of realising what an enormous power each one individually possesses of guiding the thoughts and actions of the men and women they are brought into daily contact with, and of furthering the advance of the knowledge of their profession for the good of humanity.

### NOTES FROM THE SCHOOLS.

### EXAMINATIONS IN MONTE VIDEO.

An English practitioner in Monte Video sends the following useful information as to the medical examinations in South America:—Should any anxious enquirer ever consult you as to the prospects of a young medical man in this part of the world, the following hints may be useful. The medical faculty of the university here has just published a new set of laws, in which they refuse to admit anyone to examination who is not M.B. or M.D. of a recognised university. Candidates must be well up in Spanish, in which the examinations, all *viva voce*, are conducted. They are as follows:—(1). Forty-five minutes on anatomy, physiology, general pathology, pathological anatomy, and the dissection of some part. (2). Forty-five minutes on physics, chemistry, medical natural history, biological chemistry, and the "recognition" of drugs. (3). An hour on topographical anatomy and operations, therapeutics, hygiene, and legal medicine, besides an operation on the dead body. (4) An hour on obstetrics and gynæcology, medical pathology, surgical pathology, and clinical cases, allowing a certain time for the last. The examiners are for the most part young natives, who do their best to keep out foreigners; consequently the candidate must make up his mind to spend a year in getting through, as he is sure to be spun at least once to gratify national pride (a nation which consists of some 500,000), and perhaps even rom private spleen.

We imagine that few of our readers will be very anxious to seek a diploma at Monte Video after reading the above description. That country has the further objection, at least some people will think so, that the climate is such that the vanities of tobacco and spirits have to be renounced. The correspondent in question has had to convince himself that water is the most wholesome beverage, "on the principle that everything that is nice in this world is either wrong or unwholesome." We beg to deny altogether the conclusion which this correspondent has arrived at, circumstances being, no doubt, against him, and we venture to believe if it should ever be his fate to return to the mother country that he will alter his opinion altogether, and joyfully admit that after all life is worth living in the best of all possible modes.

### EDINBURGH UNIVERSITY.

The Royal Medical Society held its opening meeting on Friday, 24th ult. at the Society's Hall, when Mr. Jonathan Hutchinson gave the Introductory address. A petition has been handed round for the students to sign, asking Professor Rutherford to publish his opening address. The Shâkow Sahib of Goudal is attending medical classes in the University.

MEN OF THE FUTURE.

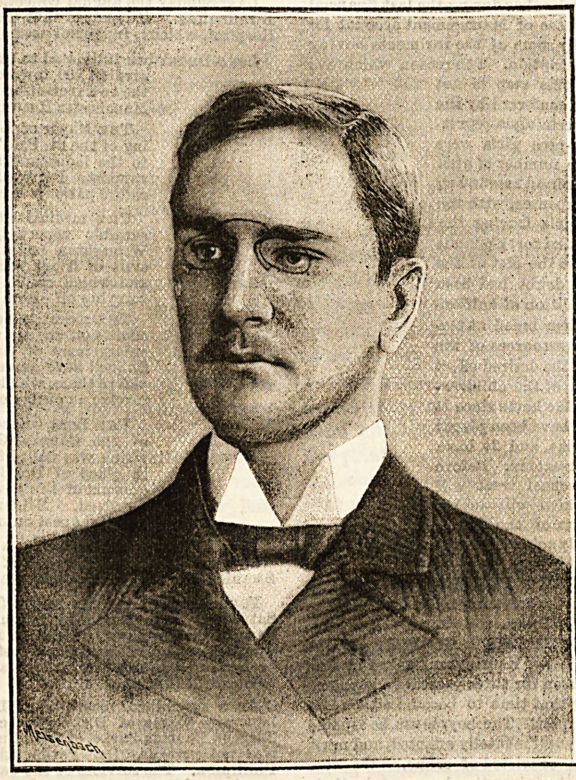

Mr. Wilfred Edgecombe (University College, Liverpool). First Class Honours in Materia Medica in Inter. M.B. London University.

### MEN OF THE FUTURE.

We give this week the portrait of Mr. Wilfred Edgecombe, who was educated at Cambridge House School, Seafort, near Liverpool. He matriculated with honours at the London University in July, 1887, and entered the medical school of University College, Liverpool, in the following October. He next passed the preliminary scientific examination of the London University in July, 1888, and has recently succeeded in getting first-class honours in materia medica and pharmaceutical chemistry, together with honours in physiology in the Intermediate M.B. Examination. We cannot do less than wish him equal success in the final examinations.

### STUDENTS' MEDICAL SOCIETIES.

### I.—Guy's Hospital Pupils' Physical Society.

A meeting was held on October 18th, when Mr. J. B. Leathes read a paper on "Tobacco." In commencing, he said the subject selected was the only one he ever ventured to discuss at the Physical Society, viz., his pipe. The controversy was a very wide one, and if science were to be the final umpire, then we were still far from a satisfactory decision. The toxic effects of nicotine were incontestable, but it was still a subject of violent controversy, whether any appreciable amount of nicotine really existed in normal tobacco smoke. The picoline and pyridine bases were however invariably present, and appeared to have a very similar action to nicotine, only less severe. An alarming picture was drawn of the disorders attributable to "smoke," but the rejoinder was that excess in anything was harmful. Mr. H. E. Durham referred to some recent work on the subject at Cambridge, and thought that nicotine could be detected in the saliva. He suggested the importance of the portal function of the liver as minimising the amount actually absorbed. He thought epithelial cancer was better explained as an inoculation, by the mouthpiece to the lip, of some virus with which the pipe became infected, owing to the careless way it was usually treated when not in the mouth, rather than as a direct toxic effect of the drug. He instanced the use of tobacco, coca, maté, &c., all over the globe as satisfying an universal need. Mr. A. J. Sharp preferred to attribute it to habit and

**Student's Medical Societies, Guy's**—*continued.*

blind imitation. He had never succeeded in extracting from any smoker any concrete reason for the habit. Possibly its regardance as one of the attributes of manhood had something to do with it. Neglecting such trifles as being asphyxiated by one's friends and expectorated upon by ones foes, if life were not too short already he would never rest till he had seen the last of a custom, which in its disgusting loathsomeness could only be considered a relic of the beastiality and hoghood of man. Mr. C. S. Pantin suggested that the particles of free carbon present might be a factor in the causation of lung disease. Mr. J. A. Howard called attention to the noxious properties of carbonic oxide gas, which was always present in tobacco smoke. Mr. V. Pendred thought the virtues of tobacco were due to the pleasurable sense of warmth derived therefrom, he had seen an Irishman smoking a piece of peat with an enjoyment it would be difficult to surpass. Mr. R. G. P. Lansdowne attributed the practice to fashion and habit, and compared the human race to a flock of sheep. Mr. R. Henderson thought it a proud moment in a young man's career when he could put a pipe in his mouth and say "I myself also am a *man*." Mr. B. W. Hogarth had no doubt that tolerance was established by practice. A man might drink or smoke for years and think he was none the worse for it, but if he met with an accident the impaired vitality of his tissues was speedily demonstrated. Mr. A. W. Sheen owed much to the germicidal properties of smoke in the dissecting room, and felt its absence acutely in the post-mortem theatre. Mr. T. Holmes had found the distraction of keeping one's pipe alight interfere seriously with the intellectual concentration essential for solid reading. Mr. F. G. Hopkins remarked that it was most difficult to detect nicotine in the presence of pyridine bases. He would have expected the distillation occurring in the bowl of a pipe to give rise to different products with different actions from those produced by the simple combustion of a cigar. Cigarettes were the most harmful form, and gave off a large percentage of carbonic oxide gas. The pleasures of life were so few and far between that the habit was quite justifiable, especially when it conduced so much to social intercourse. Messrs. L. E. Clayton and W. C. Pitt considered cigarettes the mildest form of the drug. Mr. C. F. Hogarth instanced the champion bicyclist, Osborne, as refuting the popular error that tobacco is fatal to training. We should have to give up a good many habits if we never did anything that was not dictated by considerations of health. Mr. F. J. Steward wished to know whether tobacco was really prejudicial to growth, and Mr. C. E. Carpmael suggested that smoking was prohibited in the Salvation Army from fiscal rather than sanitary motives. Mr. Leathes, in reply, attributed shortwindedness to want of Hæmoglobin and not to tobacco. If the introduction of germs on a mouthpiece must stop us smoking it was equally unsafe to eat or drink. The percentage of carbonic oxide ranged from 6 to 14 per cent., according to the conditions under which the smoke was collected. It was very striking how the plague avoided tobacconists and hard smokers. A vote of thanks to Mr. Leathes closed the meeting.

**II.—Dental Hospital Medical Society.**

This society held its first meeting on Monday, October 13th. The president (Mr. Leonard Matheson) addressed a few words of welcome to the members present, especially those who had just joined the society. To the latter he pointed out the advantages a society such as this confers on its members. There was first the knowledge gained, which was often considerable, hints being frequently obtained which proved of great use in the practical work of the profession; then the society's meetings formed a practising ground for public speaking, an acquirement which would prove of the utmost service in after years; and lastly, the meetings helped to keep alive a feeling of goodfellowship among the students of the hospital. Several interesting casual communications were brought forward by different members of the society, among the things shown being an old key instrument for extracting teeth which had been used for 50 years, and which had on one occasion removed five teeth instead of one only. This served to mark the strong advance that had been made in dental instruments. A paper was then read by Mr. W. May on the treatment of children's teeth. Among other points attention was called to the great necessity there was in modern days of early supervision of children's teeth. Things were often left too late before the little patients were taken to the dentist. Sympathy and tenderness were the great secret of success in the treatment of children, and above all things it was important never to deceive them, as irremedial harm was often done by frightening children on their first visit to the dentist and confidence lost which was never regained. After the paper had been discussed, Mr. May replied to the criticism.

**SCHOLARSHIPS AND PRIZES.—THIRD LIST.**

**St. George's Hospital.**

The following are the prize awards for the sessions 1889-90:—Pollock prize in physiology—S. Russell Wells. Ackland prize in clinical medicine—G. E. Hale. Brodie prize in clinical surgery—C. Wild. Sir C. Clarke's prize—J. W. Dickson. William Brown,

**Scholarships and Prizes**—*continued.*

£100 exhibition—H. Higgins. Treasurer's prize—G. E. Hale. Thompson medal—C. Wild. First year general proficiency prize—G. A. Clarkson. Second year general proficiency prize—H. Scott-Elliott.

**St. Mary's.**

Entrance scholarships in natural science: R. W. Dodgson, £105; J. C. Smellie, £52 10s. Exhibitions of value of £26 5s. each; J. E. P. Davies, A. T. Evans, S. P. James, in natural science; J. E. Beggs (University), in anatomy; W. Broadbent (University), in practical physiology.

**St. Thomas's.**

Entrance Scholarships in natural science: First, 125 guineas, to Walter Ernest Dixon; second, £60, to Herbert Challis Crouch, P. J. Dear qualified for the first scholarship, but is ineligible.

**University College.**

First medical entrance exhibition of £100 to F. W. Chandler; exhibition of £50 to J. H. Cook, S. Shoppee, and W. E. Dixon.

**Guy's.**

Open scholarship in arts of 100 guineas to Henry Percival Ferraby, of Bedford Modern School. Open scholarship in arts of 50 guineas, for candidates over 20 years of age, to George Frederic Still, of Caius College, Cambridge. Open scholarships n science of 125 guineas to Francis James Steward, of Old Charlton. Open scholarship in science of 50 guineas to Alfred Salter, of Lewisham.

**Schools of Surgery, Royal College of Surgeons in Ireland.**

Mayne Scholarship (£15), Thomas W. Fullerton; Carmichael Scholarship (£15), Andrew A. Gibbs; Gold Medal in Surgery, Robert B. Wright; Silver Medal, Thomas W. Fullerton; Histology, John Thrumbull; First Prize and Medal, William H. Anderson; Second Prize, Practical Pharmacy, Denis Molyneux; First Prize and Medal, Michael G. Hearne; Second Prize, Medical Jurisprudence, Catherine Maguire; First Prize and Medal, Thomas Fullerton; Second Prize, Sir P. Dun's Hospital. Mr. W. Dawson awarded the Clinical Medal and a cheque for £5.

**London Hospital.**

At the London Hospital the Entrance Science Scholarships have been awarded as follows: first, value £75, Mr. M. Cameron; second, value £50, Mr. J. Haverson. And the Buxton Scholarships: first, value £30, Mr. W. J. Mortimer; second, value £20, Mr. E. H. Cooper.

**St. Vincent's Hospital, Dublin.**

The following prizes have been awarded for the past session: Gold medal in Clinical Medicine: Edward Cosgrave. Gold medal in Clinical Surgery: Garrett A. Hickey. Special Prize: Douglas W. Wright. Junior Clinical Prize: T. C. Cummins.

**Cambridge University.**

Scholarships in Science.—Peterhouse: R. C. Slater, Clifton College, has been awarded an entrance scholarship of £40 a-year in Natural Science. St. John's: Foundation scholarships of £80, £60, and £50; minor scholarships of £50, and exhibitions of £40 and under, will be awarded at the open competition beginning on December 9th; subjects: Physics, Chemistry, General Biology, Botany, Zoology, Human Anatomy, Physiology, and Geology. Applications to be made before December 1st to the tutors, Dr. Sandys, Rev. J. T. Ward, Mr. Heitland.

### PASS LISTS.

**Society of Apothecaries of London.**

The following candidates passed the examination in the subjects indicated during September:—

Arts.—First Class: H. J. Turner, G. O. Pierce, and A. C. Turner. Second Class: J. F. Bonney, W. L. Burn, U. E. Cave, F. Copeland, E. P. Corin, F. B. Feast, G. Gocher, T. H. Guillaume, J. C. R. B. Hartnell, A. A. Hingston, C. M. Lawton, G. Morris, R. S. Ransome, F. W. Rowland, R. J. Stilwell, and J. Topham. There were 155 candidates; 87 were recorded as having passed in one or more subjects.

Surgery.—S. E. Atkins, St. Bartholomew's Hospital; H. J. L. Bullen, St. Mary's Hospital; H. S. Challenor, Cambridge University and Middlesex Hospital; J. R. Daly, Manchester; F. L. Dick, Royal Free Hospital; A. O Dornford and M. B. Dumaresq, London Hospital; C. E. M. Hey, St. Mary's Hospital; W. Hook, Westminster Hospital; E. Lambert, Yorkshire College, Leeds, and Westminster Hospital; C. P. Lovell, B.A., Oxford University and St. Thomas's Hospital; J. More, St. Bartholomew's Hospital; C. E. Reinhardt, London Hospital; L. Roberts, Cambridge University and St. Mary's Hospital; M. Royce, Royal Free Hospital.

The following candidates, having previously passed in medicine and in midwifery, were granted the diploma of the society, qualifying for registration:—

Messrs. Atkins, Hook, More, and Royce.

Medicine, Forensic Medicine, and Midwifery.—A. Allen, Charing Cross Hospital; H. J. L. Bullen, St. Mary's Hospital; H. S. Challenor, Cambridge University and Middlesex Hospital; J. R. Fuller, St. Mary's Hospital; C. E. A. MacArthur, Glasgow University; H. Sanders, Charing Cross Hospital; J. Spurr, St. Mary's Hospital.

Medicine and Forensic Medicine.—C. P. Lovell, B.A., Oxford University and St. Thomas's Hospital; C. A. Morgan, St. Thomas's Hospital.

Medicine and Midwifery.—F. C. Rogers, Victoria University, Owen's College.

**Pass Lists**—*continued.*

The subjoined candidates, having previously passed in surgery, were granted the diploma of the society, qualifying for registration:—

Messrs. Bullen, Challenor, MacArthur, Sanders, and Spurr.

**Royal Colleges of Physicians and Surgeons of Edinburgh and Faculty of Physicians and Surgeons of Glasgow.**

The quarterly examination for the Triple Qualification in Edinburgh, took place in October, with the following results:—

First Examination.—Of 45 candidates, the following 23 passed: A. A. Pim, Belfast; T. G. Johnson, Durham; J. F. D'Abren, Mangalore; J. D. Hadden, Rajahmundey; W. D. Neal, India; J. I. Twohig, Ballykerwick; J. Ewing, County Antrim; A. G. McKenna, County Galway; P. J. D. Carr, Madras; C. Kiely, Ballingarry; J. J. Uniacke, Cork; E. Wright, Dublin; D. J. Buckley, County Cork; O. L. Appleton, Newcastle on-Tyne; Eugenia Elizabeth Hannah Ure, York; J. Russell, Aberdeenshire; W. J. Roughan, Galway; G. F. Jackson, Kingstown; R. Dunlop, Belfast; R. H. Manson, County Durham; J. Featherstone, Haltwhistle; P. R. Cairns, Galashiels; and P. I. de Villiers, Cape Colony.

Second Examination.—Of 54 candidates, the following 27 passed: E. W. Allsom, Liverpool; P. Mohan, County Louth; T. F. Devane, Cork; G. R. S. Breeze, Torquay; F. Evered, Curry Rivell; J. D. Hadden, Rajahmundey; D. L. Leslie, Margate; F. W. Marsden, Moscow, Russia; C. A. Foyl, Liverpool; H. A. L. Howell, Chatham; R. Reid, Belfast; Alice Marion Umpherstone, Lasswade; Jessie MacLaren MacGregor, Edinburgh; J. J. C. Elmes, Limerick; G. M. Drury, Cork; M. Lavan, New Castle West, Limerick; D. J. MacCarthy, Riverstown; Janet Milner Campbell Gray, Bathgate; Margaret Agnes Booth, Kettering; J. F. Robertson, Edinburgh; J. Moorhead, India; C. M. Coates, Somerset; S. E. Jones, Flintshire; T. H. Lawrie, Edinburgh; B. S. Lockwood, Huddersfield; M. Gepp, Essex; and J. Grout, London.

Final Examination.—Of 102 candidates, the following 46 passed, and were admitted L.R.C.P. and S.E., and L.F.P. and S.G.: W. E. M. Wright, Bangalore; W. Johnson, Dublin; A. Wallace, Ballymena; T. J. Talbot, County Dublin; H. N. Pelly, New Brighton; J. C. Mockler, Portadown; S. T. Beckett, Liverpool; J. Wilson, Newry; H. Wellis, India; D. D. Dryden, Plymouth; L. Roberts, Conway; Mary Louisa Gordon, Liverpool; J. S. Robson, County Derry; M. Crannitch, Ballyshane; Lilian Violet Cooper, Kent; G. A. Ings, Canada; G. Wright, Canada; E. N. Féré, Canada; D. Archer, Toronto; Catherine Mary Wickham, Dublin; E. S. Leyburne, County Carlow; P. M. Sheedy, County Cork; J. Ryan, Limerick; J. Murphy, Limerick; R. E. Brown, Durham; A. E. Syme, Melbourne; S. V. R. Iyengar, Mysore; T. N. Stuart, South Africa; A. J. Murchison, Canada; F. E. Rainsford, Ballinasloe; A. McCune, Kilwinning; J. A. McDonald, County Cork; E. V. Eames, Donegal; J. H. Peet, County Kerry; G. Robertson, Leslie; F. W. Marshall, Staffordshire; A. D. C. Meade, County Cork; C. P. F. Baillieu, Victoria; T. J. Frost, County Clare; T. P. G. Wells, Blackburn; J. T. Rogers, Canada; H. M. Walker, Dublin; D. Horan, Kerry; J. C. Hodsack, Nottingham; G. Foggin, Newcastle-on-Tyne; and R. L. Moore, Belfast.

### ATHLETICS.

**Football.**

The following matches were played on October 25th:—

Caledonian v. St. Mary's Hospital.—Rain fell heavily nearly the whole time this match was in progress at Blackheath to-day (Saturday), rendering the ground in a wet and slippery condition. St. Mary's were without five of their first team, playing substitutes instead. Consequently the game was rather one-sided, the Caledonians winning by three tries to one, those for the winners being obtained by Foreman, two in the first half of the game and one in the second half. M'Caulis on each occasion took the place, but the state of the weather prevented accurate kicking.

Westminster Hospital beat Central Institute (Kennington) (R), at Raynes Park. Rain fell during most part of the game. The Hospital team played a very good combination. At the end of the game they were left victors by four goals and two tries to nil. For the winning side Lewis, Norman, Blomfield, and Moon were good; Syng, Poore, Brook, and Bloxham were good at defence.

Old Paulines beat University College Hospital (R), at Park, by two goals and a try to a try. Tries for Old Paulines by Baliozian and Kayll; goals by Yuille, one from a penalty kick; try for Hospital by Fowler.

Harlequins (Third) beat St. Bartholomew's Hospital (Third) (R), at Chiswick, by four goals and seven tries to nothing. St. Bartholomew's turning up four short, Harlequins lent two men; thirteen aside were played.

Middlesex Wanderers (Second) drew with Bartholomew's Hospital (Second), at Richmond. Each side scored a goal and a try.

Caius College, Cambridge, beat Middlesex Hospital, at Cambridge, by two goals to one. (A.)

St. George's Hospital beat St. James's by one goal and two tries to nil.

Wasps (Second) beat London Hospital (Second) by two tries to nil.

London Welsh beat Guy's Hospital, at Balham, by six tries to nil. (R.)

Royal Arsenal beat St. Bartholomew's Hospital, at Plumstead, one goal to nil. (A.)

Middlesex Hospital v. Saracens (R.)—This match was to have been played, but only the Saracens put in an appearance, so the match was postponed.

V.H.H. and H. Club.—The run on October 25th was un-

**Athletics**—*continued.*

interesting on account of the rain; only seven members started. The order of coming in was: 1, W. B. Mercer (Barts.); 2, A. N. Wilde (Barts.); 3, G. C. Hayes (King's).

### EVENTS FOR THE MONTH OF NOVEMBER.

[Items intended for this column must reach the office, 140, Strand, W.C., not later than first post on Monday mornings.]

**Medical Societies.**

St. Bartholomew's Hospital Abernethian Society.—November 6th, Dr. Tylden, "On Irregularity of the Heart"; 13th, Mr. H. Fletcher, "On Epilepsy Historically Considered"; 20th, Clinical evening; 27th, Mr. H. E. Wingfield, "On the Nature and Phenomena of Hypnotism."

Guy's Hospital Pupils' Physical Society.—November 1st, Clinical evening; 15th, Mr. T. Homes, "On Spinal Caries"; 29th, Debate "That the Medical Curriculum should be extended by one year, and that this time be devoted to pure science rather than to professional subjects," proposed by Mr. F. G. Hopkins.

St. Mary's Hospital Medical Society.—November 5th, "The London University and London Students," Mr. H. W. Page; Clinical cases, Mr. F. C. Martley; 19th, "Perithyphilitis," Mr. L. Rogers; Clinical cases, Mr. J. W. Campbell.

St. Thomas's Hospital Medical and Physical Society.—November 6th, Mr. F. Le Gros Clark, "Popular Fallacies and their Influence on Education"; 20th, Mr. W. H. C. Staverly, "Intubation."

University College Medical Society.—November 5th, Section mounting under superintendence of the Pathology and Physiology Sub-Committees; 19th, Paper by Mr. G. F. Blacker, "On Intubation of the Larynx"; Demonstrations—Pathological, Reparative Processes, Histological, Simple Glands.

Westminster Hospital Guthrie Society.—November 13th.

Irish Medical Schools and Graduates' Association.—November 25th, Autumn general meeting, at 11, Chandos Street, Cavendish Square, at five p.m., the President, Dr. George H. Kidd, in the chair.

**Athletics.**

Football Matches.

King's College.—Rugby: First Fifteen, November 1st, v. Ravens, at Wormwood Scrubbs; 8th, v. Kingston Rangers, at Bushey Park; 15th, v. St. Mary's Hospital, at Wormwood Scrubbs; 22nd, v. Catford Bridge second, at Catford Bridge; 29th, v. Lennox second, at Wormwood Scrubbs; second fifteen, 15th, v. St. Thomas's third fifteen, at Wormwood Scrubbs. Association: First eleven, 8th, v. Polytechnic Wanderers, at Wimbledon; 12th, v. Westminster Hospital, at Wormwood Scrubbs; 15th, v. St. George's Hospital, at Wormwood Scrubbs; 19th, v. R.I.E.C., at Cooper's Hill; 22nd, v. St. John's Hall, at Highbury; 29th, v. Alleyn's School, at Dulwich; second eleven, 1st, v. University College second eleven.

St. Mary's Hospital.—Rugby: First fifteen, November 1st, v. Thornton Heath, at home; 8th, v. Thurlow Park, at Streatham; 15th, Past v. Present, at home; 19th, v. Royal School of Mines, at Raynes Park; 22nd, v. Wickham Park, at home; 29th, v. Bedford, at Bedford; second fifteen, 1st, v. Crusaders, at Ealing; 8th, v. Thurlow Park second, at home: 26th, Civil Service second, at Richmond; 29th, v. Crusaders, at home. Association: 5th, v. Barnes, at Wimbledon; 8th, v. Watford Rovers, at Watford; 22nd, v. Civil Engineers, at Wimbledon; 26th, v. Woolwich, at Woolwich; 29th, v. Clapham Rovers, at Wimbledon.

Middlesex Hospital.—November 1st, v. Old Dovorians, at Balham; 8th, v. Civil Service, at Richmond; 11th, v. Epsom College, at Epsom; 15th, v. Croydon, at Croydon; 29th, v. Lennox, at Dulwich; second fifteen, 1st, v. Lewisham, at Lewisham; 15th, v. Crusaders, at Ealing; 22nd, v. Royal Naval Coll. (second), at Greenwich; 29th, v. Croydon second, at Croydon.

St. Thomas's Hospital.—First fifteen, November 1st, v. Richmond, at Richmond; 8th, v. Sandhurst, at Sandhurst; 15th, v. South Northamptonshire, at Northampton; 22nd, v. London Scottish, at Richmond; 29th, v. Rosslyn Park, at Acton. Second fifteen: 1st, v. Clapham Rovers (second), at Lambeth; 8th, v. Guy's (second), at Lambeth; 15th, v. Civil Service (second), at Lambeth; 19th, v. Barts (second), at Lambeth; 22nd, v. Middlesex Wanderers (second), at Richmond; 29th, v. Old Merchant Taylors (second), at Lambeth.

St. Bartholomew's Hospital.—First Fifteen: November 8th, v. Wickham Park, at Lee; 15th, v. R.I.E.C., at Cooper's Hill; 22nd, v. Croydon, at Croydon; 29th, v. Albion, at Devonport. Second Fifteen: November 5th, v. Royal Naval School, at Eltham; 8th, v. Marlborough Nomads, at Balham; 15th, v. Croydon, at Balham; 19th, v. St. Thomas's Hospital, at Lambeth; 22nd, v. Guy's Hospital, at Balham; 29th, v. Rosslyn Park, at Balham.

**Volunteer Corps.**

V.M.S.C., No. 1 Company.—November 1st, Chelsea Barracks, 4 p.m.; 3rd, Chenies Street, 5.15 p.m.; 6th, head-quarters, 5.15 p.m.; 10th, Chenies Street, 5.15 p.m.; 13th, head-quarters, 5.15 p.m.; 17th, Chenies Street, 5.15 p.m.

